# Factors associated with hospital admissions due to hypertension

**DOI:** 10.1590/S1679-45082018AO4283

**Published:** 2018-09-11

**Authors:** Rosimery Cruz de Oliveira Dantas, João Paulo Teixeira da Silva, Davidson Cruz de Oliveira Dantas, Ângelo Giuseppe Roncalli

**Affiliations:** 1 Universidade Federal de Campina Grande, Cajazeiras, PB, Brazil; 2 Universidade Federal do Rio Grande do Norte, Natal, RN, Brazil; 3 Universidade Federal do Rio Grande do Norte, Caicó, RN, Brazil

**Keywords:** Hospital costs, Epidemiologic factors, Hospitalization, Hypertension, Brazil, Custos hospitalares, Fatores epidemiológicos, Hospitalização, Hipertensão, Brasil

## Abstract

**Objective:**

To study the temporality of hospital admissions due to arterial hypertension and its associated factors.

**Methods:**

An ecological study with secondary data on hospital admissions due to essential arterial hypertension – ICD 10, from the Hospital Information System, the Mortality Information System and and the Primary Care Information System, between 2010 and 2015. Descriptive analysis using means, proportions and linear regression.

**Results:**

We recorded 493,299 hospitalizations due to arterial hypertension from 2010 to 2015, with an average annual progressive cost decrease of −7.76% and −24.21%. Of the patients admitted, 59.2% were women, 60.2% were non-white and 54.7% were older than 60 years. The mean length of stay was 4.2 days, and the hospitalization cost was R$307.60. The multiple linear regression variables that remained significant were the percentage of admissions due to primary care-sensitive conditions, the *per capita* income and the City Human Development Index.

**Conclusion:**

Hospital admissions due to arterial hypertension have an impact on the percentage of admissions due to primary care- sensitive conditions. Intensifying primary care activities, raising-awareness among professionals to the importance of integrated care, and investing in social development are crucial to change the reality of hypertension in terms of its control and complications.

## RESUMO

**Objetivo:**

Estudar a temporalidade das internações por hipertensão arterial e seus fatores associados.

**Métodos:**

Estudo ecológico com dados secundários referentes às internações hospitalares por hipertensão arterial essencial - CID 10, do Sistema de Informação Hospitalares, do Sistema de Informação de Mortalidade e do Sistema de Informação da Atenção Básica, no período de 2010 a 2015. Análise descritiva com média, proporção e regressão linear.

**Resultados:**

Foram registradas 493.299 internações por hipertensão arterial sistêmica de 2010 a 2015, com queda progressiva anual média de −7,76% e de −24,21% nos custos. Dentre as internações, 59,2% ocorreram em mulheres, 60,2% na raça não branca e 54,7% em maiores de 60 anos. A média de permanência foi de 4,2 dias e a do custo por internação, de R$307,60. As variáveis significativas da regressão linear múltipla foram o percentual de internações por condições sensíveis à atenção primária, a renda *per capita* e o Indice de Desenvolvimento Humano Municipal.

**Conclusão:**

As internações por hipertensão arterial sistémica impactam nos percentuais de internações por condições sensíveis à atenção primária. Intensificar as ações da Atenção Primária, sensibilizar profissionais para um cuidado integral e investir no desenvolvimento social são imprescindíveis para transformar a realidade da hipertensão no tocante ao seu controle e a suas complicações.

## INTRODUCTION

Arterial hypertension (HTN) is directly or indirectly accountable for the appearance of non-communicable chronic diseases and for a decrease in life expectancy and quality of life of individuals. It is a direct cause of hypertensive heart disease and heart failure, and is also a risk factor for conditions resulting from atherosclerosis and thrombosis, as well as for cognitive *deficits.* ^(^ ^1^ ^)^

Primary Health Care (PHC) is the preferred point of access to health services and it is where actions are taken to treat HTN. Hospital admissions due to HTN have led to the inclusion of this condition into the Brazilian list of Admissions due to primary-care sensitive conditions (APCSC). This inclusion encourages PHC workers to take measures to diagnose, treat and control HTN, which, if properly implemented, can decrease the risk of hospitalization.^(^ ^2^ ^)^

Hospitalizations lead to direct health care costs due to the use of clinical resources to produce clinical activities, expenses with patients and family members’ transportation to receive care services; and indirect costs related to loss of function and productivity resulting from the health problem.^(^ ^3^ ^)^

Between 2008 and 2012, the Health Informatics Department of the Health Care System (DATASUS - *Departamento de Informática do Sistema Único de Saúde*) recorded 5,685,827 hospital admissions due to circulatory diseases, and 479,497 of these were due to essential (primary) hypertension.^(^ ^4^ ^)^ Between 2008 and 2014, 273,393 hospital admissions of adult men with HTN were recorded, corresponding to 6.7/1,000 admissions.^(^ ^5^ ^)^

Arterial hypertension has a strong economic impact on the Brazilian Public Health Care System (SUS - *Sistema* Único *de Saúde*), standing out as a potential clinical predictor for the aggravation of other chronic conditions, increasing the length of stay and showing positive association with higher treatment costs.^(^ ^6^ ^)^

## OBJECTIVE

To study the temporality of hospital admissions due to hypertension and its associated factors.

## METHODS

An ecological study using secondary data on hospital admissions due to essential arterial hypertension, as per Revision 10 of the International Classification of Diseases and Health-Related Problems (ICD10- http://www.cid10.com.br/code), under code I10 – essential (primary) hypertension, which was the dependent variable of this study. Data were obtained from the DATASUS website Health (TABNET), epidemiology and morbidity information) in October 2016, and the search included the Hospital Information System of the SUS (SIHSUS) from 2010 to 2015, the Primary Care Information System (SIAB) and the social development indicators of the United Nations Development Program (UNDP). Of the latter, we used the Gini Coefficient, a measure of social inequality; and the City Human Development Index (CHDI), a comparative measure to rate and compare different cities based on human development and on the literacy rate among subjects aged over 18 years, calculated by city and available in the program database.

The admission rates per 10 thousand inhabitants were standardized by age and sex, as per the population of the 2010 census. For the analysis, we used the year 2012, the latest period with population-based data recorded in DATASUS. Two units were adopted for analysis: regions (general data) and cities with more than 100 thousand inhabitants for the linear regression variables. The study sample was composed of the 5,565 cities and the base-year was 2012.

The data were organized into an Excel 2013 spreadsheet and transferred to the Statistical Package of the Social Science (SPSS), version 20.0. The age was grouped into under and over 60 years; and the skin color into white and non-white (black, brown, indigenous and yellow). The descriptive statistical analysis used proportions (%), ratios (R) and means, with the respective standard deviation (SD), as a measure of central trends. The data on hospital admissions due to HTN were presented by region. The correlation between the variables was determined by the Pearson correlation, selecting based on *r*≥0.350 and p<0.2, for inclusion into the multiple linear regression, using as basis the cities with more than 100 thousand inhabitants to minimize data fluctuation and the presence of lost data.

This study is part of the doctoral thesis presented to the Graduate Program on Public Health of the *Universidade Federal do Rio Grande do Norte,* and this project was approved by the Institutional Review Board of the *Hospital Universitário Onofre Lopesunder*, under number 1.144.406, CAAE: 45926715.8.0000.5292.

## RESULTS

In Brazil, the mean of PHC indicators in 2012 was 86.85% (SD 21.38) for staff coverage (estimated population seen by the PHC teams) and, of this percentage, 57.5% of cities had 100% coverage, and only 16.9% were under 60%; 34.40% (SD 12.72) were APCSC; 20.37% (SD 81.46) were admissions due to HTN. Visits/year in *Hiperdia,* the national program for registration and follow-up of patients with hypertension and diabetes seen at the outpatient network of the SUS, reached 7,802.05 (SD 25,288.27), corresponding to the ratio of 3.7 visits per year/registered hypertensive patient. When the data were analyzed by individual city, we found variations, which depended on the city's population, the percentage coverage of PHC services, and the investment made by managers to develop the actions.

The number of clinical admissions and its evolution over time (Table 1) were the parameters used to evaluate the effectiveness of PHC actions targeted at HTN control.

**Table 1:** Evolution of hospital admissions per Brazilian region

Region	2010	2010-2012 (%)	2012	2012-2015 (%)	2015
North	9,959	10.06	10,961	-32.64	7,383
Northeast	35,112	-15.37	29,716	-8.84	27,088
Southeast	33,127	-11.52	29,310	-29.94	20,535
South	9,142	-17.73	7,521	-23.64	5,743
Mid-West	9,789	-23.97	7,443	-44.32	4,144
Brazil	97,129	-12.82	84,681	-23.37	64,893

Source: DATASUS 2016 - http://tabnet.datasus.gov.br/cgi/deftohtm.exe?sih/cnv/nrbr.def

Between 2010 and 2015, HTN accounted for 493,299 hospitalizations, *i.e.* 0.73% of total admissions. Proportionally to the population, the region with the fewest records was the Southeast, with 0.03% and 0.02% in the period. The numbers showed a linear descending behavior in the different Brazilian regions, except for the North, where they only started to decrease as of 2013. The mean decrease in hospital admissions due to HTN in Brazil was 7.76%.

The length of stay defined the severity of the case, the adequacy of care services and the costs with procedures (Table 2). The mean length of stay (4.2 days) of HTN patients decreased in the North and South regions, as compared to 2010 and 2015, but increased in all other regions. The cost of stay increased in all regions, however the total amount paid for hospitalizations decreased. Brazil had a mean cost decrease of −24.21%. The smallest decrease was seen in the Northeast and the greatest, in the Mid-West. The decrease in the mean cost of hospitalizations due to HTN was not proportional to the decrease in frequency.

**Table 2:** Length of stay and mean cost of hospitalizations due to hypertension per Brazilian region

Region	Length of stay		Cost/AIH		Mean total value paid/AIH (R$)		Difference (%)
	2010	2015	2010	2015	2010	2015	
North	3.5	3.2	223.97	227.94	2.230.517,23	1.682.881,02	-24.55
Northeast	3.8	4.3	247.88	303.44	8.703.562,56	8.219.582,72	-5.56
Sudeste	5.0	5.3	381.99	436.16	12.654.182,73	8.955.673,28	-29.23
Sul	3.3	3.2	260.90	270.88	2.385.147,80	1.555.663,84	-34.78
Centro-Oeste	3.2	3.3	251.91	256.22	2.465.946,99	1.061.775,68	-56.94
Brasil	4.0	4.3	292.98	332.37	28.456.854,42	21.568.486,41	-24.21

Source: DATASUS 2016 - http://tabnet.datasus.gov.br/cgi/deftohtm.exePsih/cnv/nrbr.def

AIH: Hospital Admission Authorization.

The distribution of admissions was based on some variables, such as sex, skin color and age range, and by region of the country (Table 3).

**Table 3:** Hospital admission due to hypertension per Brazilian region

Variable	Region	2010	%	Ratio*	2015	%	Ratio*	Difference %
Woman/Man	North	5,553	55.8	1.3:1	4,210	57.0	1.3:1	-24.2
		4,406	44.2		3,173	43.0		-28.0
	Northeast	21,839	62.2	1.6:1	16,937	62.5	1.7:1	-22.4
		13,273	37.8		10,151	37.5		-23.5
	Southeast	18,679	56.4	1.3:1	11,524	56.1	1.3:1	-38.3
		14,448	43.6		9,009	43.9		-37.6
	South	5,649	61.8	1.6:1	3,526	61.4	1.6:1	-37.6
		3,493	38.2		2,217	38.6		-36.5
	Mid-West	5,850	59.8	1.5:1	2,439	58.9	1.4:1	-58.3
		3,939	40.2		1,705	41.1		-56.7
	Brazil	57,570	59.3	1.5:1	38,636	59.5	1.5:1	-32.9
		39,559	40.7		26,255	40.5		-33.6
Skin color	North	425	6.8	1:13.6	214	5.0	1:18.8	-49.6
White/Non-white		5,806	93.2		4,025	95.0		-30.8
	Northeast	2,566	12.6	1:6.9	1,377	7.8	1:11.9	-46.3
		17,745	87.4		16,368	92.2		-7.8
	Southeast	14,646	61.1	1.6:1	8,679	55.2	1.2:1	-40.7
		9,332	38.9		7,034	44.8		-24.6
	South	6,532	87.0	6.7:1	4,193	88.0	7.3:1	-36.0
		977	13.0		571	12.0		-41.6
	Mid-west	1,740	31.3	1:2.2	746	31.6	1:2.2	-57.1
		3,826	68.7		1,616	68.4		-57.8
	Brazil	25,909	40.7	1:1.5	15,209	33.9	1:1.9	-41.3
		37,686	59.3		29,614	66.1		-21.4
<60 years	North	4,402	45.1	1.3:1	3,176	43.9	1.3:1	-27.9
≥60 years		5,364	54.9		4,060	56.1		-24.3
	Northeast	14,756	42.7	1.3:1	11,181	42.0	1.4:1	-24.2
		19,801	57.3		15,424	58.0		-22.1
	Southeast	14,699	45.0	1.2:1	8,545	42.2	1.4:1	-41.9
		17,978	55.0		11,718	57.8		-34.8
	South	4,098	45.6	1.2:1	2,430	43.0	1.3:1	-40.7
		4,893	54.4		3,216	57.0		-34.3
	Mid-west	4,651	48.0	1.1:1	1,863	45.7	1.2:1	-59.9
		5,037	52.0		2,210	54.3		-56.1
	Brazil	42,606	44.5	1:1.2	27,195	42.6	1:1.3	-36.2
		53,073	55.5		36,628	57.4		-31.0

Source: DATASUS 2016 - http://tabnet.datasus.gov.br/cgi/deftohtm.exe?sih/cnv/nrbr.def

* Ratio women:men; Ratio skin color:age group.

In 2010, in Brazil, there were 97,129 hospital admissions due to HTN, and, in 2015, 64,893, with a higher prevalence among women, with a mean ratio of 1.5 woman per man. Non-whites were more prevalent in the North, Northeast and Mid-West regions (mean R 7.6:1 in 2010 and 11.0:1 in 2015) and whites in the Southeast and South regions (mean R 1.6:1 in 2010 and 4.3:1 in 2015). The age range with the most records was over 60 years (mean R 1.2:1, between 2010 and 2015).

Missing data influenced the reality analysis. The variable skin color had 34.9% missing records in 2010, and 30.9%, in 2015, and the age range, 1.5% in 2010 and 1.6% in 2015.

The Pearson correlation was performed with variables relative to PHC, hospital and development indicators. Only the variables with a moderate to strong correlation were included in the linear regression (Table 4). The variables selected were the percentage of APCSC (r=-0.433; p<0.001), cardiologists (r=0.471; p=<0.001), illiteracy rate (r=-0.778; p<0.001), Gini coefficient (r=0.461; p<0.001), ***per capita*** income (r=0.881; p<0.001) and CHDI (r=0.881; p<0.001).

**Table 4:** Linear regression analysis of hospital admission due to hypertension

Model variables	Beta standardized	t value	p value	95%CI	
Constant	–	-4.594	<0.001	-0.039	-0.016
APCSC(%)	0.386	24.491	<0.001	0.000	0.000
Cardiologist rate	0.000	-0.008	0.994	-0.001	6.157
Illiteracy rate ≥18 years	0.033	1.062	0.288	0.000	0.000
*Per capita* income	-0.116	-3.105	0.002	0.000	0.000
Gini coefficient	-0.008	0.480	0.631	-0.005	0.008
CHDI	0.198	4.225	<0.001	0.019	0.051

95%CI: 95% confidence interval; APCSC: admissions due to primary care-sensitive conditions; CHDI: City Human Development Index. Dependent variable: hospital admission rate due to HTN/10 thousand inhabitants.

The linear regression excluded the variables relative to cardiologists, illiteracy and the Gini coefficient because they lacked statistical significance (p>0.05). The following variables were kept in the regression: percentage of APCSC and CHDI, with positive association, and negative per capita income.

## DISCUSSION

A study using a public-domain database has advantages and limitations. The advantages lie in the access to data and the diversity of indicators available. One limitation is data incompleteness, which does not preclude their use for a more in-depth long-term analysis. As for the variable hospital admissions, the limitation is similar to that found by Godoy et al.,^(^ ^7^ ^)^ with the reliability of the data restricted by their level of accuracy and completeness. Other limitations are non-recorded readmissions, lengths of stay longer than 30 days or transfers, which can lead to more than one admission record for the same user. This is due to the payment logic of the system, which favors the financial logic in detriment of epidemiology.^(^ ^8^ ^)^

Missing data in the information system leads to an epidemiological picture that does not reflect the reality. This issue can be solved through an information system that promotes communication between all systems and regards the user as a person, not a disease. Some initiatives, such as the electronic medical record and the International Primary Care Classification, must be encouraged.

Despite the inherent limitations of studies using secondary data, the information generated is valid and important and must be communicated to generate knowledge, in addition to serving as a reference for decision-making. Studying the temporality of hospital admissions due to HTN, as part of the list of APCSC, allows us to reflect on the effectiveness of PHC in controlling blood pressure, and the articulation of the different lines of care and assistance settings.

There is high variability in PHC indicators. This is due to the distinct reality of Brazilian cities, some with populations that spread far from the mean. In 2015, the coverage of PHC teams in Brazil was at 92%, thanks to the efforts and support of the Ministry of Health advocating medical care focused on health promotion and prevention. Despite the high coverage rates, the management mechanisms and social inequalities of the country allow for access to assistance and health care services to remain fragile, and their assurance depends on the reorganization of a network of health care services, supported by diagnostics, as well as specialty care and hospital services.^(^ ^9^ ^,^ ^10^ ^)^

The impact of PHC actions is clearly seen in the permanent decline in APCSC, with a rate of 31.7% in 2015. The results of investing in PHC are seen in the decreased infant mortality rates and fewer hospital admissions for preventable causes.^(^ ^9^ ^)^ Investments are surely indisputable, but it is the way they are prioritized that leads to good results.

The decline in the number of hospital admissions due to HTN is connected with the number of visits offered and the control of blood pressure levels. In this study, the ratio of visits per hypertensive patient registered with the SIAB was 3.7:1/year. This complies with the guidelines of the Ministry of Health, which recommend at least three visits per year for the proper follow-up of hypertensive patients.^(^ ^11^ ^)^ With three visits per year, professionals cannot properly monitor non-drug treatment, and end up overusing drug treatment – which is an easier path to follow. Some studies^(^ ^12^ ^,^ ^13^ ^)^ showed that users, despite taking on a positive attitude regarding drug treatment, will easily drop out, either due to inappropriate doses or incipient care.

This reality points to the need for monthly monitoring by a multidisciplinary team. However, the inclusion of professionals, in addition to the members of the basic team, is very low in PHC. This study showed that, for every 10 thousand inhabitants, there are, in average, 2.4 nutritionists, 1.98 cardiologists and 0.21 physical trainers per city. Seeking customers’ independence and individuality, and associating strategies for social mobilization towards an intersectoral effort, are the conditions required to improve issues related with health and quality of life.^(^ ^12^ ^)^

The decreased admissions due to HTN in the Brazilian regions show some distinct differences. In the North, it only started after 2013. The prevalence of risk factors, the unequal implementation and expansion of PHC and the way of organizing and operationalizing health care are determining factors of the regional differences.^(^ ^4^ ^)^ The level of awareness, the knowledge of Brazilians about the risk factors that may aggravate their health conditions, the greater PHC coverage, as well as the policies for health promotion among hypertensive patients are determining factors of the decreased number of hospitalizations.^(^ ^14^ ^–^ ^16^ ^)^

We also highlighted the encouragement of drug treatment, which, despite having beneficial effects, serves the interests of the pharmaceutical industry. The rational use of drugs has gained space with the National Drug and Pharmaceutical Care Policies, established as of the late 1990's, to prevent shortages of drugs supplied by the government,^(^ ^16^ ^)^ and is now more consistent with Ordinance 111, of January 2016, which validates medical prescriptions for 6 months, strengthening the program *Saúde Não Tem Preço* [Health is Priceless].^(^ ^17^ ^)^ This facilitates access to drugs but hinders access to monthly guidance and monitoring, increasing self-medication. To Silva et al.,^(^ ^16^ ^)^ the expansion of pharmaceutical assistance to the private sector ensures access to drugs, but not guidance and assistance, because what prompts users to go to facilities is the need to get a medical prescription for the drugs.^(^ ^18^ ^)^ Other factors that decrease hospitalizations for HTN include the wide range of anti-hypertensive drugs, the decrease in adverse effects, generic preparations and the association of drugs to promote blood pressure control in the outpatient setting.^(^ ^4^ ^)^

The length of stay showed a slight change in nearly all regions, with an increase in the average cost of hospitalizations. A study^(^ ^19^ ^)^ has pointed to a certain “normality” in this increase, specifically in the case of admissions due to hypertension, while others have shown a decreasing trend in this indicator for the same condition.^(^ ^20^ ^,^ ^21^ ^)^ The decreasing frequency of hospitalizations reflects the efficiency of PHC and health promotion policies, the expanded access of users to the services, and the greater coverage of PHC strategies.^(^ ^22^ ^,^ ^23^ ^)^ On the other hand, the decrease in costs may be a masked result of users coming into the service with HTN complications, but having their costs directed/coded to more expensive procedures, such as acute myocardial infarction and stroke.^(^ ^4^ ^)^ This is a trend in hospital services based on a procedural production logic, with a SIS/AIH (Health Information System/Hospital Admission Authorization) based on reimbursement of services provided.^(^ ^14^ ^)^

This study showed that women are hospitalized more frequently than men, which supports other studies.^(^ ^21^ ^,^ ^24^ ^)^ This was not an expected finding, since women take better care of their health and keep better control of their blood pressure than men. Other studies^(^ ^13^ ^,^ ^14^ ^)^ showed opposite results, with a linear decrease in the number of women hospitalized. This is due to them being more aware of their health condition, more present in the services and more compliant with treatment than men.^(^ ^25^ ^)^ For cultural reasons, men do not usually take care of their health or seek assistance of the Family Health Strategy.

The most prevalent age range was over 60 years, which was expected due to the deleterious effects of HTN on the body as it ages. A similar pattern was detected by VIGITEL^(^ ^15^ ^)^ and observed in the United States.^(^ ^24^ ^)^ Seniors, due to their higher load of disease and disability, end up using most of the healthcare services, which shows how current healthcare models are inefficient for this specific population.^(^ ^26^ ^)^

Non-whites were prevalent among hospitalized patients. Will et al.,^(^ ^24^ ^)^ found a greater prevalence among blacks, at the minimum ratio of 3:1, attributable to social, economic and geographic barriers, poor medical care, and the presence of comorbidities. Information on race/skin color was missing in 33% of hospitalizations, which undermines the planning and allocation of resources where real needs exist. Will et al.,^(^ ^24^ ^)^ found missing data rates of up to 22%.

The factors related to maintenance of the number of admissions due to HTN identified in the linear regression were the percentage of APCSC, the *per capita* income and the CHDI. The standardized coefficient highlights the percentage of APCSC as the variable with more impact on reducing hospitalizations due to HTN, since effective measures taken by the Family Health Strategy team can improve blood pressure control and decrease the rate of complications. The occurrence of APCSC points to disorganized offer, resolvability and access to PHC.^(^ ^27^ ^)^ The actions developed under the Family Health Strategy have led to fewer of these admissions.

Income has an influence on people's health. Its distribution directly impacts available resources as well as individual and collective infrastructure investments. Low income favors a low level of schooling, which impacts the quality of self-care and the identification of risk factors, negatively interfering with health protection behaviors.^(^ ^28^ ^)^ Therefore, it is important to invest in education which, in addition to its countless benefits, has the potential to reduce cardiovascular mortality, increase life expectancy and decrease health care costs.^(^ ^29^ ^)^

A high CHDI increases the number of hospital admissions due to HTN, due to the conditions to which individuals living in more developed cities are exposed; on the other hand, the healthcare network in these cities is better organized, with a greater offer of hospital beds to all patients requiring hospitalization. To Magnabosco et al.,^(^ ^30^ ^)^ technological evolution and salaried work contribute to increased sedentary behavior, higher intake of industrialized foods, diets higher in salt, overweight and obesity, which are factors knowingly associated with cardiovascular disease and, particularly, hypertension and its control.

It is noteworthy that, even if there was no significant association with admissions due to HTN, the PHC coverage and an expanding offer of services are some of the pillars for disseminating and strengthening integrated care. Investing in multidisciplinary teams for the Family Health Strategy as a way to dilute and share responsibilities, and intensifying health promotion and disease prevention actions are the right way towards more robust outcomes, and a subsequent decrease in hospital admissions due to HTN and its complications.

## CONCLUSION

The decrease in hospital admissions reinforces the positive results of Primary Care, as well as the need to expand access to services and integrated care, and to ensure the monitoring and follow-up of arterial hypertension by an multidisciplinary team, because this condition still generates costs to the public purse and the society.
